# Development of High Yielding Fusarium Wilt Resistant Cultivar by Pyramiding of “Genes” Through Marker-Assisted Backcrossing in Chickpea (*Cicer arietinum* L.)

**DOI:** 10.3389/fgene.2022.924287

**Published:** 2022-08-05

**Authors:** C. Bharadwaj, J. Jorben, Apoorva Rao, Manish Roorkiwal, B. S. Patil, Jayalakshmi, S. Khayum Ahammed, D. R. Saxena, M. Yasin, J. E. Jahagirdar, P. L. Sontakke, M. S. Pithia, M. K. Chudasama, Indu Swarup, R. K. Singh, S. D. Nitesh, Annapurna Chitikineni, Sarvjeet Singh, Inderjit Singh, Aditya Pratap, G. P. Dixit, A. K. Srivastava, Rajeev K. Varshney

**Affiliations:** ^1^ Division of Genetics, ICAR- Indian Agricultural Research Institute, New Delhi, India; ^2^ Center of Excellence in Genomics and Systems Biology, International Crops Research Institute for the Semi-Arid Tropics (ICRISAT), Hyderabad, India; ^3^ Genomic Breeding Lead, Khalifa Center for Genetic Engineering and Biotechnology (KCGEB), UAE University, Al Ain, United Arab Emirates; ^4^ Regional Agricultural Research Station, Kurnool, India; ^5^ RAK College of Agriculture, RVSKVV, Sehore, India; ^6^ Agricultural Research Station, Badnapur, India; ^7^ Pulse Research Station, Gujarat Agriculture University, Junagadh, India; ^8^ AICRIP on Chickpea, College of Agriculture Indore, Indore, India; ^9^ Department of Plant Breeding and Genetics, CSAUAT, Kanpur, India; ^10^ State Agricultural Biotechnology Centre, Centre for Crop and Food Innovation, Food Futures Institute, Murdoch University, Murdoch, WA, Australia; ^11^ Department of Plant Breeding and Genetics, Punjab Agricultural University, Ludhiana, India; ^12^ Crop Protection Division, Indian Institute of Pulse Research, Kanpur, India

**Keywords:** Fusarium Wilt, MABC, Pusa 391, GGE biplot analysis, recurrent parent genome recovery

## Abstract

Pusa 391, a mega *desi* chickpea variety with medium maturity duration is extensively cultivated in the Central Zone of India. Of late, this variety has become susceptible to Fusarium wilt (FW), which has drastic impact on its yield. Presence of variability in the wilt causing pathogen, *Fusarium oxysporum* f.sp. *ciceri* (*foc*) across geographical locations necessitates the role of pyramiding for FW resistance for different races (*foc* 1,2,3,4 and 5). Subsequently, the introgression lines developed in Pusa 391 genetic background were subjected to foreground selection using three SSR markers (GA16, TA 27 and TA 96) while 48 SSR markers uniformly distributed on all chromosomes, were used for background selection to observe the recovery of recurrent parent genome (RPG). BC_1_F_1_ lines with 75–85% RPG recovery were used to generate BC_2_F_1_. The plants that showed more than 90% RPG recovery in BC_2_F_1_ were used for generating BC_3_F_1_. The plants that showed more than 96% RPG recovery were selected and selfed to generate BC_3_F_3_. Multi-location evaluation of advanced introgression lines (BC_2_F_3_) in six locations for grain yield (kg/ha), days to fifty percent flowering, days to maturity, 100 seed weight and disease incidence was done. In case of disease incidence, the genotype IL1 (BGM 20211) was highly resistant to FW in Junagarh, Indore, New Delhi, Badnapur and moderately resistant at Sehore and Nandyal. GGE biplot analysis revealed that IL1(BGM20211) was the most stable genotype at Junagadh, Sehore and Nandyal. GGE biplot analysis revealed that IL1(BGM 20211) and IL4(BGM 20212) were the top performers in yield and highly stable across six environments and were nominated for Advanced Varietal Trials (AVT) of AICRP (All India Coordinated Research Project on Chickpea) in 2018–19. BGM20211 and BGM 20212 recorded 29 and 28.5% average yield gain over the recurrent parent Pusa 391, in the AVT-1 and AVT-2 over five environments. Thus, BGM20211 was identified for release and notified as Pusa Manav/Pusa Chickpea 20211 for Madhya Pradesh, Gujarat and Maharashtra, Southern Rajasthan, Bundhelkhand region of Uttar Pradesh states by the Central Sub-Committees on Crop Standards, Notification and Release of Varieties of Agricultural Crops, Ministry of Agriculture and Farmers Welfare, Government of India, for commercial cultivation in India (Gazette notification number S.O**.**500 (E) dt. 29-1-2021).Such pyramided lines give resilience to multiple races of fusarium wilt with added yield advantage.

## Introduction

Chickpea (*Cicer arietinum* L.) is a rich source of nutrition and is ranked second amongst food legumes after common bean (Bharadwaj et al., 2010). It is a self-pollinated diploid crop with genome size 740 Mbp (Varshney et al., 2013), 2n = 2x = 16 and is grown extensively in about 57 countries under varied environmental conditions. Globally it is grown in an area of 13.72 million hectares (M ha) with an annual production of 14.25 million tons (MT) (Faostat, 2020). South and South-East Asia dominate in chickpea production contributing 80% of global contribution. The largest share of chickpea production (65%, 9.0 MT) is by India followed by Australia (14%) (Merga and Haji, 2019). To attain self-sufficiency by 2050, the total pulse production in the country needs to reach 39 MT (Vision 2050; IIPR) and amongst all pulses, chickpea production alone needs to reach about 16–17.5 MT from a limited area of about 10.5 m ha with an average productivity of 15–17 q/ha (Dixit et al., 2019).

The productivity of chickpea has progressively increased from 1961, although its vulnerability to biotic and abiotic stresses has also steadily decreased, mostly because of the repeated cultivation of limited number of cultivars and use of only a few prominent donor parents in breeding programmes (Muehlbauer and Sarker, 2017). In case of biotic stresses, wilt caused by *Fusarium oxysporum* (Schlechtend.: Fr.) f. Sp. *ciceris* (Padwick) Matuo and K. Sato is a serious problem in most of the chickpea growing regions of India. The pathogen is a soil-borne, facultative fungus and leads to vascular wilting leading to an average annual yield loss of 10–30%, which can sometimes even cause complete yield loss (Sunkad et al., 2019).

Multi environmental trials are less effective to select stable genotypes across locations, especially under the effect of genotype X environment interaction (G x E x I), leading to the development of wide and specifically adaptable lines. This understanding is even more important owning to race complexity of FW in multiple-environmental trails. Management strategy for FW using chemical formulations as well as the biological control techniques is challenging as the pathogen is harbored in seed and soil. Also, cost involvement and the hazardous nature of chemicals make their use ineffective. Therefore, there is a need for cost effective management strategies which involve developing wilt resistant lines that are widely adapted (Haware and Nene, 1982; Sharma et al., 2005). Conventional breeding methodology takes a long time and is considered less effective to pyramid multiple genes conferring resistance against various races of same pathogen in a single variety. The availability of sufficient genomic information in terms of the physical map, genetic, and draft sequencing of *kabuli* chickpea genotype CDC Frontier has improved the marker-assisted backcrossing (MABC) breeding approach. Mapping studies revealed that all *foc* resistance genes (1, 3,4 and 5) are present on LG02, and tightly linked markers to these races have been identified (Millan et al., 2006; Gowda et al., 2009). Successful application of the MABC approach in chickpea for introgressing resistance/tolerance to drought (Bharadwaj et al., 2021), FW (Varshney et al., 2014a; Pratap et al., 2017), and Ascochyta blight (Varshney et al., 2014b) into popular chickpea varieties have immensely proved the application of MABC in developing elite varieties. Thus, MABC can quickly aid in developing wilt-resistant varieties and pyramid numerous genes in a single introgression line by using foreground and background selection using genome-wide SSR markers (Bharadwaj et al., 2021).This will help in developing multi-race resistant introgression lines that can be widely cultivated across the country.

Pusa 391, a mega variety that is widely grown in central India for its excellent grain quality, has become susceptible to FW since past few years and subsequently its yield had reduced drastically. Thus, in the present study, pyramiding of multiple races against *foc* 1,2,3,4 and 5 was undertaken at ICAR-Indian Agricultural Research Institute (IARI) in collaboration with International Crops Research Institute for the Semi-Arid Tropics (ICRISAT). We also report the release of Pusa Chickpea 20211, a variety developed by molecular breeding, which is highly resistant to FW and is superior to national check (JG 16).

## Materials and Methods

Pusa 391, a high yielding *desi* chickpea variety with high grain quality and market preference, was released in 1997 for Central zone of India, was chosen as the recurrent parent for introgression of FW resistance by using WR 315 as donor parent, which harbors resistant genes *foc*1, 3, 4 and 5. It has a seed size of about 20–25 g per 100 seeds, matures in 110–120 days, and has an average yield of 17–18 Q/ha (http://farmer.gov. in/imagedefault/pestanddiseasescrops/pulses.pdf). Each introgression line was planted in two replications comprising two rows of 4 m length and planted at 30 × 10 cm spacing.

### DNA Extraction and Marker Analysis

DNA was isolated from tender leaf tissues of 18–20 days old seedlings of parents, F_1_ and backcross generations, using the protocol described by Tapan et al. (2013). A total of six SSR markers, namely TA110, TA37, TR19, GA16, TA27, and TA96 reported to be in the cluster containing genes for conferring FW resistance on the linkage group CaLG02 (Millan et al., 2006) were subjected to parental polymorphism to identify polymorphic markers. PCR was carried out in the Chickpea Molecular Breeding Laboratory, Division of Genetics, ICAR-IARI using a G-STORM thermal cycler (Labtech, France). The PCR amplicons were resolved on a 1.2% agarose gel or with an ABI 3730 (Applied Biosystems). Polymorphic markers with donor and recipient cross-combinations from the hotspot region of the LG02 were used for foreground selection (Supplementary Table S1).

Based on previous studies, a panel of 365 highly polymorphic SSR markers (Thudi et al., 2011; Bharadwaj et al., 2021) were tested at the Centre of Excellence in Genomics and Systems Biology (CEGSB), ICRISAT for parental polymorphism between the donor and recurrent parents for potential use in background selection. Background selection was based on polymorphic markers identified for each donor and recipient parent cross-combination (Supplementary Table S1). Recurrent parent genome (RPG) recovery was calculated for selection using the formula suggested by Sundaram et al. (2008).

### Phenotypic Screening for Fusarium Wilt Resistance at Multiple Locations

Ten advanced MABC lines (BC_3_F_3_ progenies) were sown in wilt sick plot, along with parent Pusa 391, resistant check (WR 315), susceptible check (JG 62) and National check (JG 16) in RCBD (randomized complete block design) with two replications in the years 2017–18 across six diverse locations viz., Indore (22.7196° N, 75.8577° E), Badnapur (19.8682° N, 75.7256° E), Junagadh (21.52° N, 70.45° E) Nandyal (15.47° N, 78.48° E), Sehore (23.2032° N, 77.0844° E) and IARI-New Delhi (28°70′N, 76°58′E) during 2017-18for morphological characters such as yield (per hectare), days to 50% flowering (DFF), days to maturity (DM), and hundred seed weight (HSW) (Table 1). There was sufficient inoculum load (spore concentration of 5–6 × 10^6^ conidia/ml/g of soil) in the wilt sick plot as manifested by the 100% mortality of susceptible check JG 62. Visual observations were taken at seedling to flowering stage of crop based on mortality rate as per the classification of Sharma et al. (2019), designated as highly resistant (less than 10% plant mortality), moderately resistant (10.1–20.0% plant mortality), susceptible (20.1–40.0% plant mortality), and highly susceptible (more than 40.0% plant mortality).The superior introgression lines (IL1, IL3 and IL4) having yield superiority and reaction to FW across locations were recommend for AICRP trials. BGM20211 (IL1) and BGM 20212 (IL4) were tested in AVT-1 and AVT-2 along with checks in 2018–2019 and 2019-2020, respectively, for adaptability and yield advantage and BGM 20213 (IL 3) was recommended for AVT-1 in 2019–20.

**TABLE 1:** Mean yield- Yield performance (Kg/ha), days to Fifty percent Flowering (DFF), days to maturity (DM), 100 Seed Weight (HSW) and disease Index (DI) of chickpea at six locations during 2017–2018.

Genotype	Plant number	Yield (kg/ha)	DFF(d)	DM(d)	100SW(g)	DI (%)
IL1	P391*WR315-3	2,717.42	56.25	106.58	20.33	8.67
IL2	P391*WR315-5	2,512.25	57.33	108.08	20.23	9.93
IL3	P391*WR315-7	2,691.42	57.08	107.17	20.46	9.33
IL4	P391*WR315-9	2,705.42	57.00	107.08	20.77	8.99
IL5	P391*WR315-10	2,410.92	58.42	108.75	19.62	11.10
IL6	P391*WR315-12	2,385.42	58.92	108.75	19.77	12.18
IL7	P391*WR315-15	2,369.83	59.08	109.25	20.04	10.79
IL8	P391*WR315-17	2,474.50	57.00	109.58	20.33	10.84
IL9	P391*WR315-20	2,414.25	58.00	109.33	19.90	9.77
IL10	P391*WR315-22	2,386.17	58.33	108.83	20.14	12.11
WR 315	—	2,173.58	58.25	110.50	18.03	5.70
Pusa 391	—	1993.92	59.42	112.50	24.81	29.57
JG 16 (National Check)	—	2,274.83	57.17	112.33	18.34	17.26

% Disease incidence calculated as per Sharma et al. (2017) as,

$$\%\;Disease\;incidence=\;\frac{Total\;number\;of\;infected\;plants}{Total\;number\;of\;plants}\times 100$$

### Statistical Analysis

All analyses were performed in the R software. A preliminary variability study was conducted using *TraitStats*:R package, developed by Nitesh et al. (2021), while the GGE Biplots were performed with the metan: R package, developed by Olivoto and Lucio (2020).

## Results

### Analysis of Variance (ANOVA)

Joint ANOVA results revealed significant differences (*p* < 0.05) for all five traits among the genotypes under the study in each of the environments tested (E1, E2, E3, E4, E5, and E6) (Table 2).

**TABLE 2:** Analysis of joint variance for the trait yield (Per hectare), days to fifty per cent flowering (DFF), days to maturity (DM), 100 seed weight (100SW) and disease incidence (DI) evaluated in 13 genotypes in a different environment (**, significant at *p* ≤ 0.01; ***, significant at *p* ≤ 0.001.)

		Mean sum of square				
Sources of var	DF	Yield	DFF	DM	100SW	DI
E	5	19,252,199.94***	1808.88***	1,346.41***	113.36***	321.04***
G	12	523,189.506***	11.17***	36.75***	14.19***	6.61***
GE	60	38,466.062 ***	9.11***	15.90***	4.12**	416.05***
RESID	72	6,581.482***	1.92***	1.26***	3.05***	28.66***
CV(%)		3.34	2.40	1.03	8.72	21.52

### Foreground and Background Marker Analysis

Based on parental polymorphism, three markers viz., GA16, TA27 and TA96 were polymorphic and used for foreground selection between Pusa 391 and WR 315. For background selection, among the 365 SSR markers analysed on both parents, 141 were observed to be polymorphic. 48 of these markers that covering the whole genome uniformly, so that each chromosome contains six polymorphic markers distributed evenly were identified for background selection.

### Marker-Assisted Introgression

**FIGURE 1:**
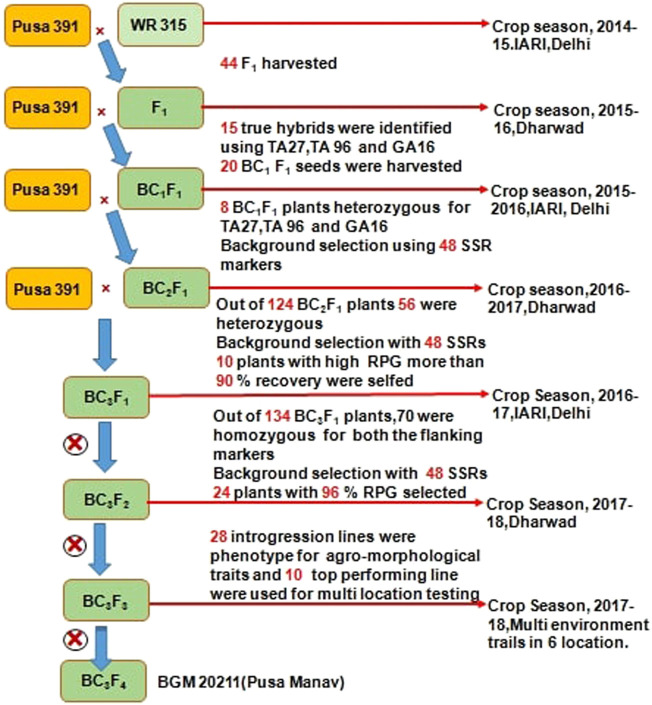
Detailed representation of marker assisted back cross bred lines of Chickpea for Fusarium wilt at ICAR-IARI.

At ICAR-IARI, New Delhi and IARI-Regional Station, Dharwad, marker-assisted backcrossing was carried out during 2014–18**.** The detailed description used for introgression for FW resistance from WR 315 to Pusa 391 is provided in Figure 1. Crosses were carried out between Pusa 391 (recurrent parent) and WR 315 (donor) in crop season 2014-15 to develop F1s. A total of 44 F_1_s were obtained, out of which 40 germinated and 15 were confirmed as true hybrids using polymorphic foreground markers (TA27, TA96, GA16). These F_1_s were utilized to make backcross with Pusa 391, and 20 BC_1_F_1_ seeds were generated during the off-season in 2015–16 at Dharwad. Out of 20 BC_1_F_1_ seeds produced, eight positive plants for foreground marker and had the higher genome recovery of 75–85% were selected for backcrossing to generate 124 BC_2_F_1_ seeds during the main season IARI, New Delhi in 2015–16. From the 124 BC_2_F_1_ plants, 56 were heterozygous for foreground markers, of which ten plants with more than 90% genome recovery were used for generation of backcrossing at IARI regional station Dharwad to generate 134 BC_3_F_1_.Then upon foreground selection, 70 plants were observed to be heterozygous. These 70 plants were subjected for background selection using 48 SSR markers, and 24 BC_3_F_1_ plants showing more than 96% RPG recovery were selected and selfed. Finally, with two rounds of selfing, 28 best BC_3_F_3_ plants were analysed with foreground and background markers with 90–97% RPG recovery and showing agronomic superiority were selected. The top ten high yielding, highly disease resistant lines (<10% disease incidence) with plant type similar to Pusa 391 (recurrent parent) were evaluated at multiple locations.

### Phenotypic Performance of Pusa 391 MABC Lines in Multi-Location Trails

Introgression lines (BC_3_F_3_) were phenotyped at six diverse environments, Indore, Badnapur, Junagadh, Nandyal, Sehore and IARI-New Delhi, in the crop season 2017–2018 and transgressive segregants were identified by morphological superiority and with high wilt resistance (Table 1). IL1, IL4, and IL3 were the top performers in the multi-location trial with a mean yield advantage of 20, 19 and 18%, respectively, over the national check JG 16 and mean yield gain of 36, 35 and 35% over recurrent parent Pusa 391. Disease incidence index over multiple locations of IL1, IL2, IL3, IL4, and IL 9 were 8.66, 9.93, 9.33, 8.99 and 9.76, respectively, which showed a highly resistant reaction. On the other hand, IL5, IL6, IL7, IL8 and IL10 were 11.1, 12.1, 10.8, 10.8 and 12.1 respectively, which showed moderately resistant reactions.

The hundred seed weight (HSW) of IL1 (BGM 2011) across locations was 20.33 g, IL4 (BGM 20212) was 20.77, and IL3 (BGM 20213) was 20.46, as compared to recurrent parent Pusa391, which recorded 24.81 g IL1 (BGM 20211) had early flowering (56 days) and early maturity (107days) compared to the parent Pusa 391 flowered in 59 days and matured in 112 days. Variation in genotype for different traits studied is presented as descriptive statistics in Table 3. IL1 (BGM 20211) and IL4 (BGM 20212) were the top two performers in multilocation trials conducted, and they were nominated for AICRP trials in 2018–19 (AVT-1) and 2019-20 (AVT-2). The next top performer, IL3 (BGM 20213), was nominated for AICRP trials in 2019–20 (AVT-1).

**TABLE 3:** Descriptive statistics involving Maximum (Max), Mean, Minimum (Min), standard deviation (Sd) and Standard error (Se).

Variable	Max	Mean	Min	Sd	Se
DFF	78	57.87	43	7.95	0.64
DMM	124	109.13	93	7.28	0.58
DI	57	12.02	2.1	7.53	0.6
100 GW (UNIT)	26.9	20.14	14.8	2.79	0.22
Yield (UNIT)	3,902	2,423.82	1,082	822.19	66.04

### National Advanced Varietal Trial Evaluation

Advanced varietal trial data of BGM 20211 and BGM 20212 indicated that they were early flowering (51 and 53 days, respectively) as well as early maturing (106 and 107 days, respectively) lines. Under ICAR-AICRP on chickpea, special trials were conducted in known as “Wilt resistance introgression lines” (WRIL) trials that can assess lines performance in multiple locations. There was a 23 and 35% overall weighted percentage increase in AVT-1 and AVT-2 respectively with overall mean of 29% over recurrent parent Pusa 391. In the case of IL2 (BGM 20212), the overall weighted percentage increased over the mean of 28.5%, with 24% in AVT1 and 33% in AVT 2, respectively, over the recurrent parent Pusa 391 (Tables 4, 5). Disease incidence in multiple locations as per AICRP trials are presented in Supplementary Table S2.

**TABLE 4:** AVT-1 Comparison of yield (kg ha−1) performance of BGM 20211 and BGM 20212 introgression line developed in the genetic background of Pusa 391, with the recurrent genotype at six locations in the Advanced Varietal Trials−1 of ICAR–All India Coordinated Research Project on Chickpea conducted during 2018–2019 (Source: AICRP Chickpea Annual Report 2018–2019).

Entry	Indore	Junagadh	Sehore	Badnapur	Nandyal	Mean	Freq^a^
BGM 20211	2078	3,526	1,671	1,584	1,131	1998	5/5
BGM 20212	2,156	3,718	1,659	1,437	1,121	2018.2	5/5
Pusa 391(Recurrent parent	1,648	3,064	1,123	1,337	961	1,626.6	—
Critical difference at 5%	75	370	114	188	301	—	—
CV(%)	2.7	7.9	5.9	9.5	16.7	—	—
General mean (kg/ha)	1921	3,197	1,319	1,358	1,237	1806.4	—
state avg. yield (kg/ha)	1,165	1,244	1,165	782	1,051	1,081.4	—

a Frequency, the ratio of a number of locations in which the introgression line performs higher than the recurrent parent to the total number of locations evaluated.

**TABLE 5:** AVT-2 Comparison of yield (kg ha−1) performance of BGM 20211 and BGM 20212 introgression line developed in the genetic background of Pusa 391, with the recurrent genotype at six locations in the Advanced Varietal Trials−2 of ICAR–All India Coordinated Research Project on Chickpea conducted during 2019–2020 (Source: AICRP Chickpea Annual Report 2019–2020).

Entry	Indore	Junagadh	Sehore	Badnapur	Nandyal	Mean	Freq^a^
BGM 20211	2000	3,915	2,182	2,343	1,428	2,373.6	5/5
BGM 20212	2,162	3,699	2,148	2,136	1,539	2,336.8	5/5
Pusa 391	1,643	3,286	1,530	1,494	826	1755.8	—
Critical difference at 5%	368	598	142	473	263	—	—
CV(%)	13.7	11.8	6	16.7	12	—	—
General mean (kg/ha)	1854	3,507	1,649	1963	1,513	2097.2	—
State avg. Yield (kg/ha)	1,165	1,244	1,165	782	1,051	1,081.4	—

a Frequency, the ratio of number of locations in which the introgression line performs higher than the recurrent parent to the total number of locations evaluated.

GGE biplot analysis: GGE biplot analysis explained 66.11% of the total variation, where PC1 (wilt incidence) and PC2 (resistance stability) accounted for 51.64 and 14.47% variation, respectively.

1) Mean vs. Stability

**FIGURE 2:**
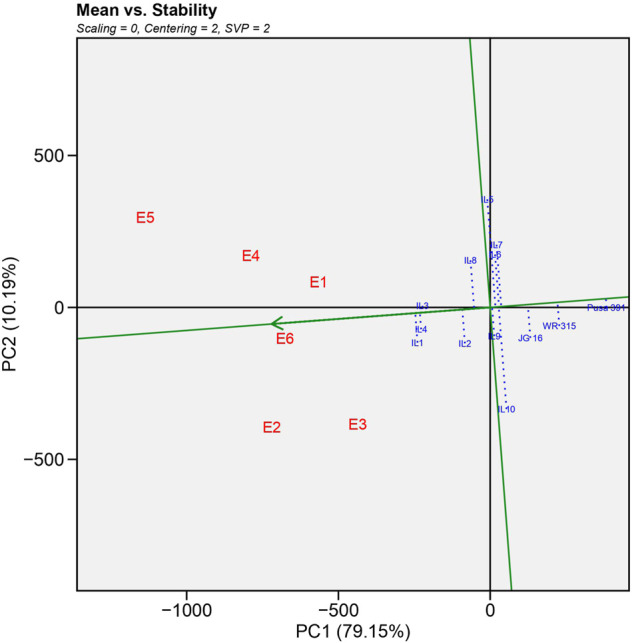
Average-environment coordination (AEC) view showing the mean yield performance and stability of different genotypes based on genotype and genotype interaction (GGE)-biplot analysis.

As illustrated in Figure 2, GGE biplot analysis explained a total of 89.34% variation, the horizontal axis (PC1) accounted for 79.15% of the total variation. It represented the main effect of genotypes, whereas the vertical axis (PC2) accounted for 10.19% of total variation and showed the impact of G X E interaction (Yan and Kang, 2002). The average environment coordinate (AEC) axis is a single arrow line passed from the biplot origin to the average environment, depicted by a dotted circle. On the vertical axis, ILs located to the right of the AEC indicated higher yield than average yield and vice versa. Thus, the biplot organized the yield performance as IL1>IL4>IL3>IL2>IL8 in that order. However, the recurrent parent Pusa 391 and national check JG 16 showed a lower yield than ILs. The AEC vertical axis displayed the stability of genotype yield, which was considered stronger if the horizontal AEC axis line length was shorter (Lakew et al., 2014; Harish et al., 2020). The IL1, IL4 and IL3 were the most stable and high yielding as they were farthest from origin and shortest vector length.

2) Which-Won-Where pattern analysis

**FIGURE 3:**
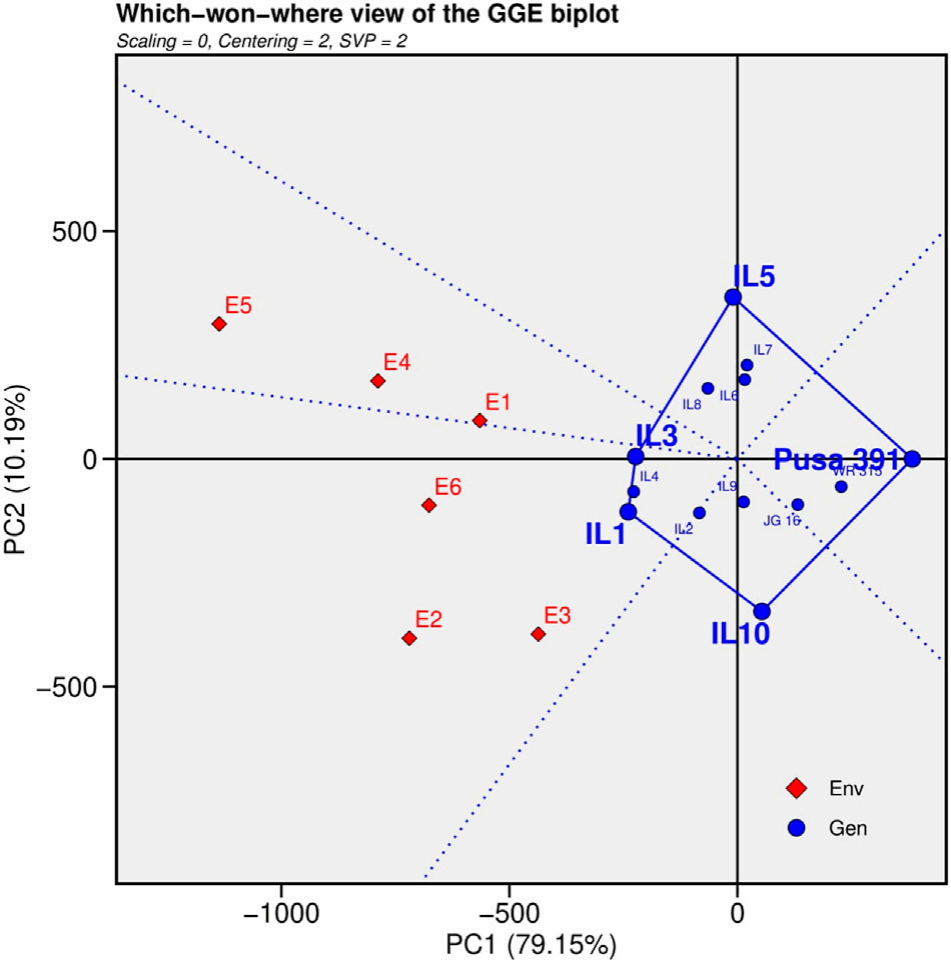
“Which-won-where” view for the primary component of interaction (PC1) and second principal component (PC 2).

The polygon view was generated by interconnecting the markers of the ILs that were farthest from the biplot origin with straight lines, resulting in markers of all cultivars being contained in the polygon (Figure 3). To divide the biplot into various sectors, lines perpendicular to each side of the polygon or their extensions were drawn from the biplot origin. The peak cultivar in each sector was the top cultivar for traits found in that section; on the other hand, genotypes found inside the polygon and near the biplot’s origin were not sensitive to changing environmental conditions (Dimitrios et al., 2008).The genotypes positioned at the greatest distance from the biplot origin were the best or worst ILs in particular or every environment. IL1 performed superior under E2 (Junagadh), E3 (Sehore) and E6 (Nandyal) and IL3 performed superior in E1 (Indore), E4 (Badnapur) and E5 (New Delhi) from the which won where pattern analysis.

3) Evaluation of testing locations based on discrimination power vs. representativeness

**FIGURE 4:**
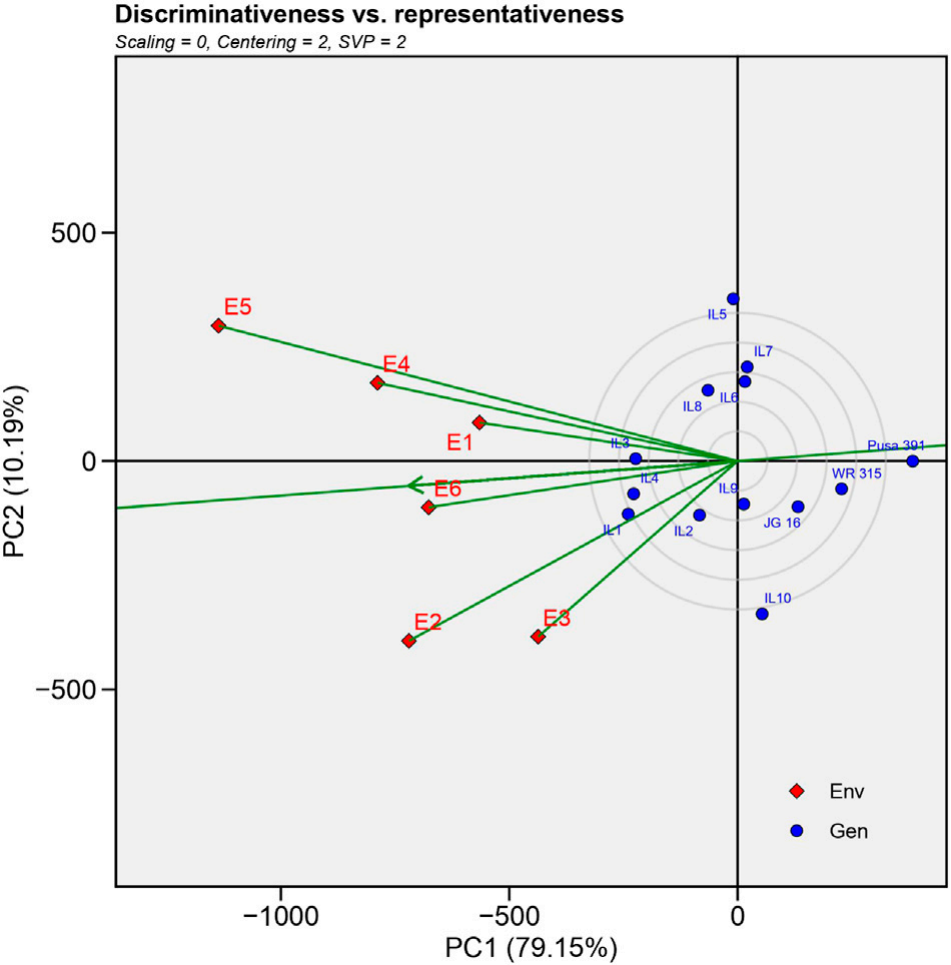
GGE-biplot environment view for yield that shows the correlation between test environments and correlation coefficient between any two environments is approximated by the cosine angle between their vectors.

An ideal location needs to be highly distinctive and represent the target location simultaneously (Yan, 2010). The ability of a place to maximise the diversity among potential cultivars in a study is referred to as discrimination (Blanche and Myers, 2006). The ability to represent, reveals that the study included an environment that was indicative of the conditions in the other locations (Mohammadi and Amri, 2012). An ideal environment will identify genotypes with high and stable yield. The small circle in the GGE-biplot display represents a perfect position determined by the mean coordinates of all testing locations (Figure 4). There was a positive association between the length of the location vector and the ability to discriminate between locations, but a negative correlation between the angle of the location vector with the ideal location and the location’s representativeness of the target environment (Yan, 2010). The observed angle between E1, E4, E5 and E2, E3, E6 was less than 90^0^, indicating a positive correlation among environment sets, and similar results can be expected in these regions. Following analysis, it was observed that E5 (New Delhi)>E2>E3>E4>E6>E1 had the longest environmental vectors among the test environments, making it the most “discriminating location” with the potential to distinguish different genotypes. The ranking of environments in terms of being the best representative locations was E6>E1>E4>E2,E3, E5 were in the order, and thus E6 (Nandyal) can be considered the most representative environment.

## Discussion

Fusarium wilt has been a widely distributed disease that can cause upto 100% yield loss based on varietal susceptibility and changing climatic conditions that have resulted in the shift of large chickpea growing area from cool long Northern India to warm short central and southern India (Patil et al., 2015). The presence of eight physiological races of *foc* (0,1A,1B/C, 2, 3, 4, 5 and 6) has been reported across different countries (Haware and Nene, 1982). FW is prevalent in dry and warm semi-arid tropic (SAT) regions of Asia, Africa and South America (Nene and Sheila, 1996). Race 1 is typical in Central and Peninsular India, race 2 in Northern India and 3 and 4 in Punjab and Haryana (Haware et al., 1992). Also, some of the cultivars are susceptible with time, which could be attributed to variability in wilt incidence and genetic differences among genotypes and genotype x environment interactions (Sharma et al., 2012).

The “5Gs” breeding technique (genome assembly, germplasm characterization, gene function identification, genomic breeding, and gene editing) has recently been proposed for obtaining precision and boosting crop improvement to satisfy future demands for nutritious food (Varshney et al., 2019). MABC using genome-wide SSR markers for foreground and background selection for recovery of recurrent parent genome is an environment-independent, precise, and rapid strategy for developing disease-resistant cultivars (Bharadwaj et al., 2021). Deploying resistant variety is one of the key sustainable strategies that breeders can adopt as it is most effective and environmentally safe for integrated disease management (Sharma et al., 2017).

Genetic inheritance studies reveal that resistance to race 0,1A,2 and 4 are either digenic or trigenic, but races 3 and 5 were monogenic (Tullu et al., 1999; Tekeoglu et al., 2000).Based on several inter- and intra-specific crosses, it was reported that wilt resistance genes *foc*1, *foc*3, *foc* 4 and *foc* 5 (Races 1, 3, 4 and 5) are mapped on two gene clusters, i.e., GA16 and TA96 (*foc* 1 and 4 clusters), TA 96 and TA 27 (*foc* 3 and 5 clusters) (Millan et al., 2006). However, resistance genes *per se* and proteins that were reported to be involved in pathogen defense were localized in between the region or in close vicinity of the gene cluster. Also, resistance loci *ar1* and *ar2a* against Ascochyta blight were localized on LG02 and near *foc* gene clusters. Thus, LG02 is considered a hot spot for pathogen defense (Millan et al., 2006).

The major hindrance in chickpea breeding for FW is variation in pathogen races over multiple locations and their interaction with different weather conditions over space and time (Sharma et al., 2014). Stable high yielding lines with high disease resistance are required to develop widely adaptable varieties. (Srivastava et al., 2021). Thus, GGE Mean vs. Stability analysis recorded IL1>IL4>IL3 were most stable and high yielding introgression line in the order and the worst performing genotype was Pusa 391 > WR 315 > JG16. Which-won-where analysis revealed IL1 performed best under Junagadh, Sehore and Nandyal region and IL3 performed best in Indore, Badnapur and New Delhi from the which-won-where plot. The New Delhi environment was considered the most discriminating location because this location is subjected to distinct climatic conditions compared to other environments. Discrimination and representativeness analyses reveal that E6 (Nandyal) is the most representative location. The genotypes were highly stable in this location, as was reported by Sharma et al. (2019) while screening for wilt-resistant genotypes in wilt sick plot over ten locations. The top three best performing introgression lines (IL1, IL4 and IL3) with more than 30% yield advantage over recurrent parent Pusa 391 were nominated for AVT trials based on the multi-location studies.

MABC approach was used to pyramid races1, 2, 3,4 and 5 for FW and RPG recovery. In the current study, BC_2_F_1_ and BC_3_F_1_ generation achieved 90 and 96% RPG recovery for selected MABC lines in the genetic background of Pusa 391. Similar genome recovery was reported by Mannur et al., 2019and Bharadwaj et al., 2021 in chickpea. Thus, MABC reduces the time taken to develop a variety and such genome recovery is usually possible inBC_4_F_1_ and more generations in conventional breeding.

The introgression line IL1 (BGM 20211) is highly resistant to FW and moderately resistant to stunt, collar rot, dry root, pod borer and possesses excellent grain, colour and shape, as per AICRP report, 2020. The grain protein content was found to be 18.92%. In the case of 100 SW, parent Pusa 391 had a higher 100 SW than introgression line BGM 20211. Also, it is an early flowering and early maturing IL (57 days to flowering and 107 days to maturity), that can fit in the double cropping system and is ideal for the sustainability of the rice-wheat cropping system (Bharadwaj et al., 2021).Further, it can also escape heat stress at harvest in central India compared to Pusa 391, which matures in 112 days.

In the case of disease incidence, BGM 20211 was highly resistant for FW in Junagadh, Indore, IARI-New Delhi and found moderately resistant at Sehore and Nandyal. BGM 20212 was highly resistant in Junagarh and New Delhi and moderately resistant in Indore, Sehore and Nandyal. BGM 20213 was highly resistant in Junagarh, Indore, New Delhi and found to be moderately resistant at Sehore and Nandyal. National check (JG 16) was moderately resistant in Junagarh, Indore, New Delhi and Nandyal and susceptible in Sehore. Also, recurrent parent Pusa 391 was moderately resistant in Badnapur, susceptible in New Delhi, Nandyal, Junagarh and Indore, highly susceptible in Sehore. Superior performance across locations confirms the molecular basis of pyramiding with morphological and wilt sick studies.

In the case of FW, introgression for *foc*1 (Varshney et al., 2014a), *foc*2 (Pratap et al., 2017) and *foc* 4 (Mannur et al., 2019) were developed on the elite genetic background of C214, Pusa 256 and Annigeri, respectively. Mannur et al., 2019 reported a 125% mean yield advantage of superior introgression line over the recurrent parent JG 74 and Super Annigeri 1 reported a8% mean yield advantage over recurrent parent Annigeri 1. In our study, BGM 20211 outperformed parent Pusa 391 by 29% (average over five regions in AVT-1 and AVT-2) and national check (JG16) by 28% (average over 6 locations in multi-location trials), which include southern India (Nandyal), Central India (Badnapur, Indore and Junagarh) and North India (New Delhi).This variety profusely branches and possesses a large number of pods per unit area and has demonstrated an overall weighted mean yield of 2,186 kg/ha and the highest yield potential reported was 3,915 kg/ha in a wilt stress environment, compared to 1,691 kg/ha in case of recurrent parent Pusa 391.

This is the first report in pulses where FW genes are pyramided in recurrent parent background and released for commercial cultivation using the MABC approach.Marker assisted back cross breeding approach was utilized for pyramiding of FW resistance for different races (*foc* 1,2,3,4 and 5). Foreground selection was performed using three SSR markers (GA16, TA 27 and TA 96) and background selection for recovery (RPG) of recurrent parent genome was done using 48 SSR markers that were uniformly distributed on all chromosomes. Multi-location evaluation of advanced introgression lines (BC_2_F_3_) was done in six locations for grain yield parameters and wilt screening along with GGE biplot analysis. IL1 (BGM 20211) and IL4 (BGM 20212) were the top performers in yield and were highly stable across all environments and were nominated for Advanced Varietal Trials (AVT) of AICRP (All India Coordinated Research Project on Chickpea). BGM 20211 was identified for release and notified as Pusa Manav for Madhya Pradesh, Gujarat and Maharashtra states by the Central Sub-Committees on Crop Standards, Notification and Release of Varieties of Agricultural Crops, Ministry of Agriculture and Farmers Welfare, Government of India, for commercial cultivation in India. High yielding pyramided lines for FW are important to avoid economic losses and for improving Chickpea production across India.

## Data Availability

The original contributions presented in the study are included in the article/Supplementary Material, further inquiries can be directed to the corresponding authors.
