# Supplementary material for: Development of High Yielding Fusarium Wilt Resistant Cultivar by Pyramiding of “Genes” Through Marker-Assisted Backcrossing in Chickpea (Cicer arietinum L.)
Source: Front Genet. 2022 Aug 5;13:924287. doi: 10.3389/fgene.2022.924287 (PMC9388742; doi:10.3389/fgene.2022.924287)
Supplement: Supplementary file 1 [file Table1.DOCX]

**Supplementary Table 1. Markers (SSR) used in the foreground selection and background selection for Pusa Chickpea Manav (BGM 20211)**

| **FOREGROUND MARKERS** | | | | | |  |
| --- | --- | --- | --- | --- | --- | --- |
| **Marker Name** | **LG** | **Recurrent parent allele**  **(P-391) bp** | | | **Donor Parent Allele**  **(WR 315) bp** | **WRIL**  **BGM**  **20211 allele bp** |
| GA 16 | 2 | 267 | | | 220 | 220 |
| TA 96 | 2 | 293 | | | 280 | 280 |
| TA 27 | 2 | 223 | | | 240 | 240 |
| **BAKGROUND MARKERS** | | | | | |  |
| **Marker Name** | **LG** | | **Recurrent parent allele**  **(P-391) bp** | **Donor Parent Allele**  **(WR 315) bp** | | **WRIL**  **BGM**  **20211 allele bp** |
| NCPGR 136 | 1 | | 153 | 166 | | 166 |
| NCPGR 184 | 1 | | 160 | 154 | | 160 |
| TR 43 | 1 | | 339 | 330 | | 339 |
| TR 56 | 1 | | 238 | 226 | | 238 |
| TA 113 | 1 | | 192 | 215 | | 192 |
| TA 140 | 1 | |  |  | |  |
|  |  | |  |  | |  |
| GA 20 | 2 | | 184 | 202 | | 184 |
| TA 103 | 2 | | 168 | 171 | | 168 |
| H2110 | 2 | | 180 | 160 | | 180 |
| TAA60 | 2 | | 160 | 172 | | 160 |
| NCPGR 144 | 2 | | 217 | 220 | | 217 |
| GAA 47 | 2 | | 178 | 185 | | 178 |
|  |  | |  |  | |  |
| GA 119 | 3 | | 264 | 273 | | 264 |
| STMS 28 | 3 | | 298 | 264 | | 298 |
| TR 2 | 3 | | 277 | 261 | | 277 |
| TA 64 | 3 | | 297 | 306 | | 297 |
| NCPGR 103 | 3 | | 220 | 200 | | 220 |
| TA 76 | 3 | | 230 | 250 | | 230 |
|  |  | |  |  | |  |
| TA 2 | 4 | | 211 | 218 | | 211 |
| ICCM 249 | 4 | | 167 | 161 | | 167 |
| NCPGR 21 | 4 | | 150 | 143 | | 150 |
| NCPGR 164 | 4 | | 237 | 231 | | 237 |
| NCPGR 33 | 4 | | 248 | 240 | | 248 |
| NCPGR 129 | 4 | | 280 | 272 | | 280 |
|  |  | |  |  | |  |
| TAA 58 | 5 | | 301 | 277 | | 277 |
| TA 71 | 5 | | 187 | 195 | | 187 |
| TS 43 | 5 | | 225 | 230 | | 225 |
| TA 25 | 5 | | 215 | 206 | | 215 |
| TR 56 | 5 | | 220 | 210 | | 220 |
| TR 59 | 5 | | 180 | 173 | | 180 |
|  |  | |  |  | |  |
| TA 14 | 6 | | 277 | 289 | | 277 |
| TA 22 | 6 | | 211 | 288 | | 211 |
| TA 176 | 6 | | 239 | 334 | | 239 |
| NCPGR 4 | 6 | | 287 | 296 | | 287 |
| TA 80 | 6 | | 250 | 238 | | 250 |
| TR 40 | 6 | | 167 | 175 | | 167 |
|  |  | |  |  | |  |
| TA 18 | 7 | | 210 | 200 | | 210 |
| TAA 55 | 7 | | 148 | 156 | | 148 |
| NCPGR 130 | 7 | | 230 | 240 | | 230 |
| TR 117 | 7 | | 184 | 170 | | 184 |
| TA 28 | 7 | | 212 | 221 | | 212 |
| TA 21 | 7 | | 260 | 254 | | 260 |
|  |  | |  |  | |  |
| GA 6 | 8 | | 225 | 219 | | 225 |
| TS 45 | 8 | | 241 | 244 | | 241 |
| NCPGR 170 | 8 | | 232 | 240 | | 232 |
| NCPGR 50 | 8 | | 197 | 208 | | 197 |
| TA 118 | 8 | | 164 | 178 | | 164 |
| NCPGR 19 | 8 | | 232 | 236 | | 232 |
| **Total number of loci fixed for recurrent parent allele (A type)** | | | | | | **48** |
| **Total number of loci fixed for donor parent allele (B type)** | | | | | | **3** |
| **Total** | | | | | | **51** |
| **Percent recurrent parent genome (RPG) recovery including half of the allele frequency at heterozygous loci** | | | | | | **94** |

Supplementary Table 2a: Comparison of disease incidence among different genotypes across locations in the Advanced Varietal Trials−I of ICAR–All India Coordinated Research Project on Chickpea conducted during 2018–2019 (Source: AICRP Chickpea Annual Report 2018–19)

| Entry | Indore | Junagadh | Sehore | Badnapur | Nandyal |
| --- | --- | --- | --- | --- | --- |
| BGM 20211 | 19.5 | 2.1 | 14.6 | 12.5 | 36.1 |
| BGM 20212 | 9.6 | 3.8 | 7.69 | 0 | 12.5 |
| Pusa 391(Recurrent parent | 9.8 | 29.8 | 6.67 | 20.83 | 32.5 |
| Check(S) | 100 | 100 | 100 | 100 | 100 |
| Check(R) | 8.7 | 6.3 | 5.52 | 0 | 4.25 |
| LSI% | 20.4 | 6.9 | 20.6 | 35.8 | 21.12 |

Supplementary Table 2b: Comparison of disease incidence among different genotypes across locations in the Advanced Varietal Trials -II of ICAR–All India Coordinated Research Project on Chickpea conducted during 2019–2020 (Source: AICRP Chickpea Annual Report 2019–20)

| Entry | Indore | Junagadh | Sehore | Badnapur | Nandyal |
| --- | --- | --- | --- | --- | --- |
| BGM 20211 | 34.4 | 3.85 | 14.7 | 0 | 9.17 |
| BGM 20212 | 47.3 | 2.65 | 11.1 | 5.41 | 8.96 |
| Pusa 391(Recurrent parent) | 10 | 17.79 | 65.4 | 3.48 | 45.5 |
| Check (S) | 100 | 100 | 49.3 | 100 | 100 |
| Check (R) | 7 | 10.95 | 5.8 | 0 | 4.25 |
| LSI% | 52.1 | 11.8 | 52.2 | 21.98 | 16.48 |
